# Crystal structure of the tetra­meth­yl(pheneth­yl)cyclo­penta­dienylmolybdenumtricarbonyl dimer

**DOI:** 10.1107/S2056989018008885

**Published:** 2018-06-26

**Authors:** Loren Brown, Danny Marron, Casey Smith, Joseph Merola

**Affiliations:** a Virginia Tech, Dept. of Chemistry 0212, Blacksburg, VA, 24060, USA

**Keywords:** crystal structure, metal dimer, cyclo­penta­dienyl ligand, carbonyl complex, molybdenum

## Abstract

The structure of the dimer bis­{tricarbon­yl[η^5^-tetra­meth­yl(pheneth­yl)cyclo­penta­dien­yl]molybdenum}(*Mo*—*Mo*), [Mo_2_(C_17_H_21_)_2_(CO)_6_], at 102 K has triclinic (*P* ) symmetry. The reaction between tetra­meth­yl(pheneth­yl)cyclo­penta­diene and molybdenum hexa­carbonyl in refluxing xylenes for 18 h led to a 56% yield of the dimer as a red solid. The asymmetric unit of the structure is the tetra­meth­yl(pheneth­yl)cyclo­penta­dienylmolybdenumtricarbonyl moiety and the entire dimeric mol­ecule is generated by inversion symmetry. The Mo—Mo bond length is 3.2773 (3) Å, a value slightly above the mean value for all [CpMo(CO)_3_]_2_ compounds listed in the CSD and slightly below the mean for [Cp*Mo(CO)_3_]_2_ complexes.

## Abstract 2

The structure of the dimer bis­{tricarbon­yl[η^5^-tetra­meth­yl(pheneth­yl)cyclo­penta­dien­yl]molybdenum}(*Mo*—*Mo*), [Mo_2_(C_17_H_21_)_2_(CO)_6_], at 102 K has triclinic (*P* 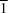) symmetry. The Mo—Mo bond length is 3.2773 (3) Å, a value slightly above the mean value for all [CpMo(CO)_3_]_2_ compounds listed in the CSD and slightly below the mean for [Cp*Mo(CO)_3_]_2_ complexes.

## Chemical context   

Following the discovery of ferrocene in 1951 (Werner, 2012 ▸), the cyclo­penta­dienyl (Cp) ligand became ubiquitous in studies of sandwich and half-sandwich compounds. As a result of the high reactivity of the C—H bond in the cyclo­penta­dienyl ligand in some circumstances, penta­methyl­cyclo­penta­dienyl (Cp*) soon became a common replacement for Cp. In recent years, researchers have begun investigating Cp-type ligands with mixed substitution of the ring. The cyclo­penta­dienyl ligand ranges from unsubstituted, Cp, monomethyl substituted, Cp’, other non-fully substituted, Cp^*R*^, and fully methyl­ated, Cp*. The most systematically studied ring substitution is the tetra­meth­yl(*R*)cyclo­penta­dienyl (Cp*^R^) ligand where *R* represents any group other than methyl.
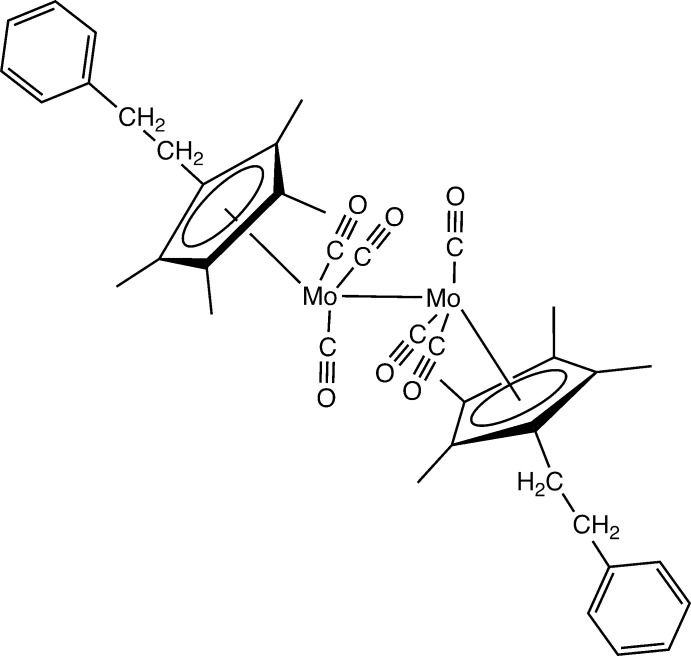


**Figure 1:**
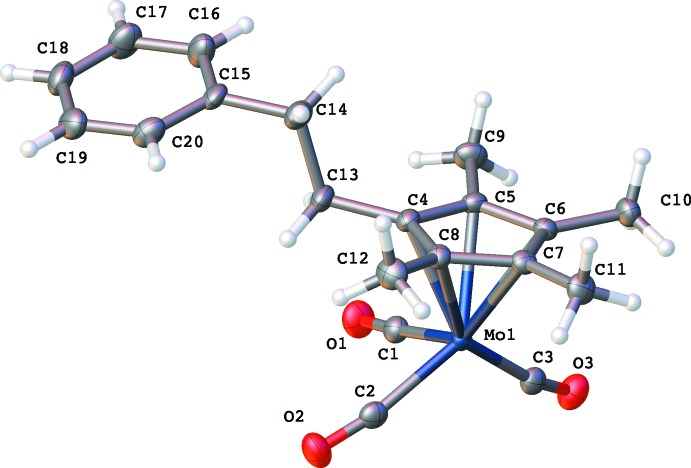
The asymmetric unit of the title compound, showing the labeling scheme. The displacement ellipsoids are shown at the 70% probability level.

Our group (DuChane *et al.*, 2018 ▸; Brown *et al.*,2016 ▸) and others (Piou *et al.*, 2017 ▸) have examined various Cp*^R^ ligands in rhodium and iridium chemistry. Perhaps one of the more intriguing of the metal systems studied with Cp, Cp^*R*^, Cp*, and Cp*^R^ ligands is that of molybdenum hexa­carbonyl. Reaction between Cp ligands and Mo(CO)_6_ leads to the formation of the dinuclear [CpMo(CO)_3_] types of complexes. Reaction between Cp*^R^ ligands and Mo(CO)_6_ has been studied systematically in various laboratories, including reports on the structures of a variety of [Cp*^R^Mo(CO)_3_]_2_ compounds. In this report, we add to the structural descriptions of the range of [Cp*^R^Mo(CO)_3_]_2_ compounds with the addition of the complex with *R* = phenethyl (Fig. 1 ▸).

## Structural commentary   

**Figure 2:**
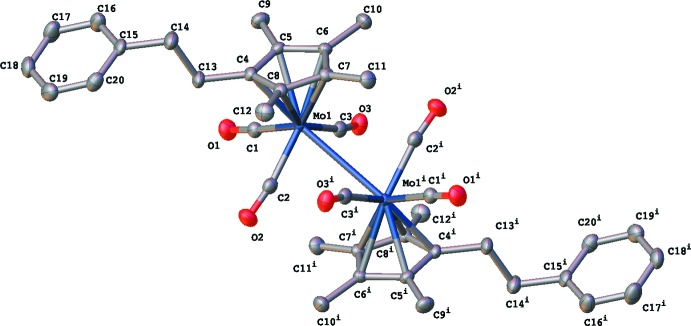
The complete mol­ecular unit of the title compound. The displacement ellipsoids are shown at the 70% probability level. Hydrogen atoms are omitted for clarity. Symmetry code: (i) 2 − *x*, 1 − *y*, 1 − *z*.

The η^5^-tetra­methy(pheneth­yl)lcylo­penta­dienylmolyb­denum­tricarbonyl dimer (Fig. 2 ▸) crystallizes in space group *P* 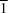 with the η^5^-tetra­meth­yl(pheneth­yl)cyclo­penta­dienylmolybdenum­tri­carb­onyl moiety being the asymmetric unit and with the entire dimer being generated by an inversion center. The Cp*^R^ ligands are in a transoid arrangement about the Mo—Mo bond with that bond being 3.2773 (3) Å in length. The disposition of the phenethyl groups on the Cp*^R^ rings can best be described by measuring the torsion angle made by the two Mo atoms, the C atom on the ring to which the phenethyl group is attached and the attaching C atom of the phenethyl group (Mo1^i^—Mo1—C5—C10). For the title compound, this angle is 119.59 (10)°.

Other structural features of note are the Mo—C—O angles. Two of the CO ligands of the dimer point away from the Mo—Mo bond and are close to linearity with an Mo—C2—O2 (Mo′—C2′—O2′) angle of 176.27 (14)°. The four CO ligands that point over the Mo—Mo bond have angles of 167.87 (14)° and 171.42 (14)°. Further commentary on these values can be found in the *Database* s*urvey* section.

## Database survey   

There are a number of molybdenum tricarbonyl dimers in the CSD database (Version 5.39, last update May 2018: Groom *et al.*, 2016 ▸) with cyclo­penta­dienyl and substituted cyclo­penta­dienyl ligands. The database was searched using the program *Conquest* (Bruno *et al.*, 2002 ▸) and the data was analyzed with the program *Mercury* (Macrae *et al.*, 2008 ▸). The structure of the completely methyl­ated [Cp*Mo(CO)_3_]_2_ complex was first determined by Clegg and co-workers (GAVKUJ; Clegg *et al.*, 1988 ▸). Examining all types of cyclo­penta­dienyl ligands (Cp, Cp^*R*^, Cp* and Cp*^R^, along with other unique substitution patterns), the mean Mo—Mo distance is 3.252 Å, ranging from a low of 3.211 to a high of 3.307 Å. The low end of the scale is comprised of unsubstituted or singly substituted Cp ligands and the high end of Cp* and Cp*^R^ ligands. For this latter group, the Mo—Mo distances range from a minimum of 3.256 Å to a maximum of 3.307 Å with a mean distance of 3.286 Å. Within this range, the title compound is at the lower end, slightly below the average. The most extensive series of [Cp*^R^Mo(CO)_3_]_2_ compounds were made and structurally characterized in the laboratories of Lin and co-workers. These include *R* = ethyl, propyl, butyl and cyclo­hexyl (LEXROX, GEVBAM, LALNAP, LEXFUR; Ma *et al.*, 2013 ▸, 2010 ▸) as well as aryl and substituted-aryl substituents *R* = *p*-bromo­phenyl, *p*-tolyl and *p*-meth­oxy­phenyl (DUFKEW, HENKUZ, HENDIO; Dong *et al.*, 2015 ▸; Ma *et al.*, 2013 ▸) Complexes with the benzyl (TULLAO; Ma *et al.*, 2009 ▸) and with the 2-pyridyl­methyl side chain (OGIHAP; Ma *et al.* 2015 ▸) were also structurally characterized.

Nearly all of the relevant structures in the database have the transoid arrangement of the Cp rings across the Mo—Mo bond. An exception was found in the work of Gould, Barker and co-workers in which they found the cisoid isomer of [CpMo(CO)_3_]_2_ (CYPMOC01) as a minor product in their attempt to prepare a different Mo compound (Gould *et al.*, 1988 ▸).

The Mo—C—O angles for all of the compounds in the database show the same pattern as for the title compound with the carbonyl ligands lying over the Mo—Mo bond, bent back from linearity by between 9 and 15°.

## Supra­molecular features   

The nature of the weak hydrogen bond, especially C—H⋯*X* hydrogen bonds, has garnered considerable inter­est over the last two decades given the cumulative influence that many such weak bonds can have on a crystal structure (Desiraju, 2002 ▸). The distinction between C—H⋯O hydrogen bonds and simple van der Waals inter­actions, a matter (not an insignificant one) of analyzing bond distances and angles, will not be discussed here. However, there are quite a few short C—H⋯O inter­actions in the crystal of the title compound (Table 1 ▸). The shortest ones are *intra*molecular interactions between H12*C* and O3^i^ (H12*C* ^i^ and O3) and between H10*A* and O2^i^ (H10*A* ^i^ and O2) *viz.* 2.47 and 2.54 Å, respectively). These are on the short end of H—O distances found in the literature, but deviate considerably from linearity. These inter­actions involve the oxygen atoms on the carbonyl ligands that point over the Mo—Mo bond. More importantly in terms of supra­molecular features, the short inter­molecular C—H⋯O distances are found between O1, the oxygen atom on the carbonyl ligand that points up and away from the Mo—Mo bond, and H12*B* on a neighboring mol­ecule at a distance of 2.62 Å (C—H⋯O angle 141°). The inter­action between the aromatic ring and a bound CO may be more important as it is closer to linear: C20—H20⋯O3^iii^ (2.65 Å and 167°). These inter­molecular C—H⋯O inter­actions and others knit the dimers into bilayers that extend in the *ab* plane. The C—H⋯O inter­actions are confined to the middle of the bilayer; only van der Waals inter­action exist between the bilayers. Based on the literature, both the intermolecular and intramolecular C—H⋯O inter­actions appear to be on the stronger end of weak inter­actions. (Steiner & Desiraju, 1998 ▸; Taylor, 2016 ▸). Their classification as ‘hydrogen bonds’ awaits more complete analysis of all compounds of this type.

## Synthesis and crystallization   

Under an atmosphere of pre-purified nitro­gen, 0.5892 g (2.60 mmol) of [2-(2,3,4,5-tetra­methyl­cyclo­penta-2,3-dien-l -yl)eth­yl]benzene and 0.6852 g (2.60 mmol) of molybdenum hexa­carbonyl were dissolved in 10 ml of xylenes and refluxed for 18 h. At the end of 18 h, xylenes were removed under reduced pressure and purified on a column of alumina using a 1:1 di­chloro­methane:hexa­nes solvent system. Following removal of solvents, 0.6096 g of the [Cp*^R^Mo(CO)_3_]_2_, *R* = phenethyl, (55.8% yield) was isolated. The bulk material was shown to be the desired compound based on: NMR, ^1^H, 400 MHz, C_6_D_6_): δ 1.82 (*s*, 12H, 4 sets of CH_3_), 1.90 (*s*, 12H, 4 sets of CH_3_), 2.58–2.72 (*m*, SH, 4 sets of CH_2_), 7.04–7.26 (*m*, 10H, phen­yl) p.p.m. IR in CH_2_Cl_2_: υ = 1914 (*st*), 1898 (*st*) and 1856 (*st*) cm^·1^.

A portion of the product was dissolved in CH_2_Cl_2_ and the solvent was allowed to evaporate slowly, yielding crystals suitable for X-ray crystallography.

## Refinement   

Crystal data, data collection and structure refinement details are summarized in Table 2 ▸. H atoms were included in calculated positions and treated as riding: C—H = 0.95–0.99 Å with *U*_iso_(H) = 1.5*U*_eq_(C-methyl) and 1.5*U*_eq_(C) for other H atoms.

## Supplementary Material

Crystal structure: contains datablock(s) I. DOI: 10.1107/S2056989018008885/pj2053sup1.cif

Structure factors: contains datablock(s) I. DOI: 10.1107/S2056989018008885/pj2053Isup2.hkl

Supporting information file. DOI: 10.1107/S2056989018008885/pj2053Isup3.mol

CCDC reference: 1841068

Additional supporting information:  crystallographic information; 3D view; checkCIF report

## Figures and Tables

**Table 1:** C—H⋯O interactions (Å, °)

*D*—H⋯*A*	*D*—H	H⋯*A*	*D*⋯*A*	*D*—H⋯*A*
C10—H10*A*⋯O2^i^	0.98	2.54	3.277 (2)	132
C12—H12*B*⋯O1^ii^	0.98	2.62	3.432 (2)	141
C12—H12*C*⋯O3^i^	0.98	2.47	3.127 (2)	124
C20—H20⋯O3^iii^	0.95	2.65	3.579 (2)	167

Symmetry codes: (i) 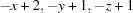; (ii) 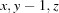; (iii) 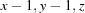.

**Table 2:** Experimental details

Crystal data	
Chemical formula	[Mo_2_(C_17_H_21_)_2_(CO)_6_]
*M*_r_	810.61
Crystal system, space group	Triclinic, *P* 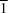
Temperature (K)	102
*a*, *b*, *c* (Å)	8.2123 (3), 8.7728 (3), 13.4788 (3)
α, β, γ (°)	91.005 (2), 96.975 (2), 115.515 (3)
*V* (Å^3^)	867.24 (5)
*Z*	1
Radiation type	Mo *K*α
μ (mm^−1^)	0.77
Crystal size (mm)	0.38 × 0.21 × 0.08

Data collection	
Diffractometer	Rigaku OD Xcalibur Eos Gemini ultra
Absorption correction	Analytical [*CrysAlis PRO* (Rigaku OD, 2018 ▸), based on expressions derived by Clark & Reid (1995 ▸)]
*T*_min_, *T*_max_	0.680, 0.890
No. of measured, independent and observed [*I* > 2σ(*I*)] reflections	27828, 5873, 5362
*R*_int_	0.044
(sin θ/λ)_max_ (Å^−1^)	0.754

Refinement	
*R*[*F* ^2^ > 2σ(*F* ^2^)], *wR*(*F* ^2^), *S*	0.028, 0.058, 1.06
No. of reflections	5873
No. of parameters	221
H-atom treatment	H-atom parameters constrained
Δρ_max_, Δρ_min_ (e Å^−3^)	0.53, −0.44

Computer programs: *CrysAlis PRO* (Rigaku OD, 2018 ▸), *SHELXT* (Sheldrick, 2015*a* ▸), *SHELXL2018* (Sheldrick, 2015*b* ▸) and *OLEX2* (Dolomanov *et al.*, 2009 ▸).

## supplementary crystallographic information

### Crystal data

**Table:**

[Mo_2_(C_17_H_21_)_2_(CO)_6_]	*Z* = 1
*M**_r_* = 810.61	*F*(000) = 414
Triclinic, *P*1	*D*_x_ = 1.552 Mg m^−^^3^
*a* = 8.2123 (3) Å	Mo *K*α radiation, λ = 0.71073 Å
*b* = 8.7728 (3) Å	Cell parameters from 13703 reflections
*c* = 13.4788 (3) Å	θ = 3.8–32.0°
α = 91.005 (2)°	µ = 0.77 mm^−^^1^
β = 96.975 (2)°	*T* = 102 K
γ = 115.515 (3)°	Irregular, red
*V* = 867.24 (5) Å^3^	0.38 × 0.21 × 0.08 mm

### Data collection

**Table:**

Rigaku OD Xcalibur Eos Gemini ultra diffractometer	5873 independent reflections
Radiation source: fine-focus sealed X-ray tube, Enhance (Mo) X-ray Source	5362 reflections with *I* > 2σ(*I*)
Graphite monochromator	*R*_int_ = 0.044
Detector resolution: 8.0061 pixels mm^-1^	θ_max_ = 32.4°, θ_min_ = 3.8°
ω scans	*h* = −12→12
Absorption correction: analytical [CrysAlis PRO (Rigaku OD, 2018), based on expressions derived by Clark & Reid (1995)]	*k* = −12→12
*T*_min_ = 0.680, *T*_max_ = 0.890	*l* = −20→20
27828 measured reflections	

### Refinement

**Table:**

Refinement on *F*^2^	Primary atom site location: dual
Least-squares matrix: full	Hydrogen site location: inferred from neighbouring sites
*R*[*F*^2^ > 2σ(*F*^2^)] = 0.028	H-atom parameters constrained
*wR*(*F*^2^) = 0.058	*w* = 1/[σ^2^(*F*_o_^2^) + (0.022*P*)^2^ + 0.3543*P*] where *P* = (*F*_o_^2^ + 2*F*_c_^2^)/3
*S* = 1.06	(Δ/σ)_max_ = 0.001
5873 reflections	Δρ_max_ = 0.53 e Å^−^^3^
221 parameters	Δρ_min_ = −0.44 e Å^−^^3^
0 restraints	

### Special details

**Table:**

Geometry. All esds (except the esd in the dihedral angle between two l.s. planes) are estimated using the full covariance matrix. The cell esds are taken into account individually in the estimation of esds in distances, angles and torsion angles; correlations between esds in cell parameters are only used when they are defined by crystal symmetry. An approximate (isotropic) treatment of cell esds is used for estimating esds involving l.s. planes.

### Fractional atomic coordinates and isotropic or equivalent isotropic displacement parameters (Å^2^)

**Table:**

	*x*	*y*	*z*	*U*_iso_*/*U*_eq_	
Mo1	0.93816 (2)	0.48286 (2)	0.61194 (2)	0.00956 (4)	
O1	0.78895 (17)	0.71295 (16)	0.70709 (9)	0.0220 (3)	
O2	0.56781 (16)	0.37365 (16)	0.46973 (9)	0.0222 (3)	
O3	1.24069 (16)	0.85014 (15)	0.60601 (9)	0.0204 (2)	
C1	0.8417 (2)	0.6281 (2)	0.66857 (12)	0.0147 (3)	
C2	0.7106 (2)	0.4198 (2)	0.51564 (12)	0.0151 (3)	
C3	1.1275 (2)	0.7122 (2)	0.59670 (12)	0.0145 (3)	
C4	0.8288 (2)	0.30540 (19)	0.73685 (11)	0.0130 (3)	
C5	1.0051 (2)	0.43723 (19)	0.77681 (11)	0.0130 (3)	
C6	1.1381 (2)	0.41714 (19)	0.72621 (11)	0.0118 (3)	
C7	1.0452 (2)	0.27264 (19)	0.65532 (11)	0.0126 (3)	
C8	0.8539 (2)	0.20336 (19)	0.66167 (11)	0.0124 (3)	
C9	1.0464 (2)	0.5607 (2)	0.86552 (12)	0.0190 (3)	
H9A	1.145490	0.669669	0.854880	0.028*	
H9B	1.083160	0.516547	0.926130	0.028*	
H9C	0.937299	0.576264	0.873419	0.028*	
C10	1.3404 (2)	0.5136 (2)	0.75498 (12)	0.0177 (3)	
H10A	1.401889	0.501161	0.699594	0.027*	
H10B	1.379416	0.468997	0.814706	0.027*	
H10C	1.372132	0.633737	0.769440	0.027*	
C11	1.1345 (2)	0.1950 (2)	0.59372 (12)	0.0171 (3)	
H11A	1.061270	0.153498	0.527506	0.026*	
H11B	1.143810	0.100409	0.627383	0.026*	
H11C	1.256743	0.280489	0.586017	0.026*	
C12	0.7090 (2)	0.0385 (2)	0.61166 (13)	0.0189 (3)	
H12A	0.596077	0.049941	0.592218	0.028*	
H12B	0.687155	−0.050467	0.658256	0.028*	
H12C	0.749102	0.008481	0.551819	0.028*	
C13	0.6516 (2)	0.2734 (2)	0.77284 (12)	0.0170 (3)	
H13A	0.552247	0.223631	0.715620	0.020*	
H13B	0.656572	0.383359	0.795769	0.020*	
C14	0.6044 (2)	0.1545 (2)	0.85895 (13)	0.0202 (3)	
H14A	0.580824	0.038789	0.834467	0.024*	
H14B	0.708000	0.195660	0.914158	0.024*	
C15	0.4371 (2)	0.1511 (2)	0.89656 (12)	0.0163 (3)	
C16	0.4522 (2)	0.2522 (2)	0.98076 (13)	0.0224 (4)	
H16	0.568229	0.314413	1.019772	0.027*	
C17	0.3000 (2)	0.2640 (2)	1.00913 (14)	0.0246 (4)	
H17	0.312472	0.332842	1.067395	0.029*	
C18	0.1313 (2)	0.1754 (2)	0.95232 (14)	0.0216 (3)	
H18	0.027715	0.185143	0.970506	0.026*	
C19	0.1133 (2)	0.0722 (2)	0.86855 (13)	0.0213 (3)	
H19	−0.003018	0.010151	0.829742	0.026*	
C20	0.2644 (2)	0.0593 (2)	0.84142 (12)	0.0189 (3)	
H20	0.250509	−0.012951	0.784518	0.023*	

### Atomic displacement parameters (Å^2^)

**Table:**

	*U*^11^	*U*^22^	*U*^33^	*U*^12^	*U*^13^	*U*^23^
Mo1	0.00936 (6)	0.01083 (6)	0.00941 (6)	0.00498 (4)	0.00246 (4)	0.00076 (4)
O1	0.0232 (6)	0.0229 (6)	0.0247 (6)	0.0143 (5)	0.0046 (5)	−0.0032 (5)
O2	0.0170 (6)	0.0279 (7)	0.0217 (6)	0.0112 (5)	−0.0018 (5)	−0.0027 (5)
O3	0.0196 (6)	0.0162 (6)	0.0231 (6)	0.0047 (5)	0.0070 (5)	−0.0009 (5)
C1	0.0126 (7)	0.0154 (7)	0.0149 (7)	0.0048 (6)	0.0024 (5)	0.0018 (6)
C2	0.0181 (7)	0.0167 (7)	0.0131 (7)	0.0096 (6)	0.0046 (6)	0.0004 (6)
C3	0.0151 (7)	0.0170 (7)	0.0141 (7)	0.0092 (6)	0.0043 (5)	0.0009 (6)
C4	0.0124 (7)	0.0152 (7)	0.0124 (7)	0.0061 (6)	0.0040 (5)	0.0049 (5)
C5	0.0150 (7)	0.0156 (7)	0.0097 (6)	0.0075 (6)	0.0027 (5)	0.0027 (5)
C6	0.0117 (6)	0.0137 (7)	0.0105 (6)	0.0062 (5)	0.0007 (5)	0.0014 (5)
C7	0.0145 (7)	0.0132 (7)	0.0129 (7)	0.0086 (6)	0.0021 (5)	0.0021 (5)
C8	0.0121 (7)	0.0119 (6)	0.0137 (7)	0.0053 (5)	0.0024 (5)	0.0027 (5)
C9	0.0236 (8)	0.0224 (8)	0.0125 (7)	0.0117 (7)	0.0020 (6)	−0.0015 (6)
C10	0.0118 (7)	0.0219 (8)	0.0171 (7)	0.0057 (6)	0.0004 (6)	0.0016 (6)
C11	0.0186 (7)	0.0183 (7)	0.0187 (8)	0.0118 (6)	0.0041 (6)	−0.0002 (6)
C12	0.0181 (8)	0.0137 (7)	0.0212 (8)	0.0038 (6)	0.0011 (6)	0.0006 (6)
C13	0.0139 (7)	0.0222 (8)	0.0175 (7)	0.0088 (6)	0.0075 (6)	0.0074 (6)
C14	0.0195 (8)	0.0265 (9)	0.0203 (8)	0.0132 (7)	0.0098 (6)	0.0097 (7)
C15	0.0147 (7)	0.0190 (7)	0.0163 (7)	0.0066 (6)	0.0080 (6)	0.0075 (6)
C16	0.0143 (7)	0.0288 (9)	0.0185 (8)	0.0041 (7)	0.0030 (6)	−0.0016 (7)
C17	0.0239 (9)	0.0284 (9)	0.0189 (8)	0.0083 (7)	0.0072 (7)	−0.0040 (7)
C18	0.0167 (8)	0.0251 (9)	0.0256 (9)	0.0092 (7)	0.0104 (7)	0.0073 (7)
C19	0.0146 (7)	0.0224 (8)	0.0208 (8)	0.0023 (6)	0.0025 (6)	0.0042 (7)
C20	0.0212 (8)	0.0174 (8)	0.0158 (7)	0.0055 (6)	0.0059 (6)	0.0000 (6)

### Geometric parameters (Å, º)

**Table:**

Mo1—Mo1^i^	3.2773 (3)	C10—H10B	0.9800
Mo1—C1	1.9536 (16)	C10—H10C	0.9800
Mo1—C2	1.9943 (16)	C11—H11A	0.9800
Mo1—C3	1.9764 (16)	C11—H11B	0.9800
Mo1—C4	2.3065 (15)	C11—H11C	0.9800
Mo1—C5	2.3076 (14)	C12—H12A	0.9800
Mo1—C6	2.3737 (14)	C12—H12B	0.9800
Mo1—C7	2.4135 (14)	C12—H12C	0.9800
Mo1—C8	2.3767 (15)	C13—H13A	0.9900
O1—C1	1.1532 (19)	C13—H13B	0.9900
O2—C2	1.1526 (19)	C13—C14	1.546 (2)
O3—C3	1.1579 (19)	C14—H14A	0.9900
C4—C5	1.439 (2)	C14—H14B	0.9900
C4—C8	1.431 (2)	C14—C15	1.510 (2)
C4—C13	1.502 (2)	C15—C16	1.387 (2)
C5—C6	1.425 (2)	C15—C20	1.397 (2)
C5—C9	1.502 (2)	C16—H16	0.9500
C6—C7	1.432 (2)	C16—C17	1.395 (3)
C6—C10	1.499 (2)	C17—H17	0.9500
C7—C8	1.434 (2)	C17—C18	1.380 (3)
C7—C11	1.500 (2)	C18—H18	0.9500
C8—C12	1.500 (2)	C18—C19	1.388 (3)
C9—H9A	0.9800	C19—H19	0.9500
C9—H9B	0.9800	C19—C20	1.384 (2)
C9—H9C	0.9800	C20—H20	0.9500
C10—H10A	0.9800		
			
C1—Mo1—Mo1^i^	123.14 (5)	C11—C7—Mo1	128.87 (10)
C1—Mo1—C2	79.57 (6)	C4—C8—Mo1	69.55 (8)
C1—Mo1—C3	77.69 (6)	C4—C8—C7	107.68 (13)
C1—Mo1—C4	87.41 (6)	C4—C8—C12	125.14 (14)
C1—Mo1—C5	84.52 (6)	C7—C8—Mo1	73.99 (8)
C1—Mo1—C6	115.39 (6)	C7—C8—C12	126.49 (14)
C1—Mo1—C7	142.19 (6)	C12—C8—Mo1	129.29 (10)
C1—Mo1—C8	120.77 (6)	C5—C9—H9A	109.5
C2—Mo1—Mo1^i^	74.11 (5)	C5—C9—H9B	109.5
C2—Mo1—C4	101.17 (6)	C5—C9—H9C	109.5
C2—Mo1—C5	135.42 (6)	H9A—C9—H9B	109.5
C2—Mo1—C6	152.82 (6)	H9A—C9—H9C	109.5
C2—Mo1—C7	120.20 (6)	H9B—C9—H9C	109.5
C2—Mo1—C8	94.42 (6)	C6—C10—H10A	109.5
C3—Mo1—Mo1^i^	68.01 (5)	C6—C10—H10B	109.5
C3—Mo1—C2	112.68 (6)	C6—C10—H10C	109.5
C3—Mo1—C4	139.37 (6)	H10A—C10—H10B	109.5
C3—Mo1—C5	103.97 (6)	H10A—C10—H10C	109.5
C3—Mo1—C6	93.26 (6)	H10B—C10—H10C	109.5
C3—Mo1—C7	116.21 (6)	C7—C11—H11A	109.5
C3—Mo1—C8	150.35 (6)	C7—C11—H11B	109.5
C4—Mo1—Mo1^i^	146.34 (4)	C7—C11—H11C	109.5
C4—Mo1—C5	36.34 (5)	H11A—C11—H11B	109.5
C4—Mo1—C6	59.42 (5)	H11A—C11—H11C	109.5
C4—Mo1—C7	58.65 (5)	H11B—C11—H11C	109.5
C4—Mo1—C8	35.55 (5)	C8—C12—H12A	109.5
C5—Mo1—Mo1^i^	145.99 (4)	C8—C12—H12B	109.5
C5—Mo1—C6	35.42 (5)	C8—C12—H12C	109.5
C5—Mo1—C7	58.51 (5)	H12A—C12—H12B	109.5
C5—Mo1—C8	59.37 (5)	H12A—C12—H12C	109.5
C6—Mo1—Mo1^i^	110.63 (4)	H12B—C12—H12C	109.5
C6—Mo1—C7	34.79 (5)	C4—C13—H13A	108.6
C6—Mo1—C8	58.58 (5)	C4—C13—H13B	108.6
C7—Mo1—Mo1^i^	94.24 (4)	C4—C13—C14	114.48 (13)
C8—Mo1—Mo1^i^	110.83 (4)	H13A—C13—H13B	107.6
C8—Mo1—C7	34.83 (5)	C14—C13—H13A	108.6
O1—C1—Mo1	176.27 (14)	C14—C13—H13B	108.6
O2—C2—Mo1	171.42 (14)	C13—C14—H14A	109.9
O3—C3—Mo1	167.87 (14)	C13—C14—H14B	109.9
C5—C4—Mo1	71.87 (8)	H14A—C14—H14B	108.3
C5—C4—C13	125.92 (14)	C15—C14—C13	108.93 (13)
C8—C4—Mo1	74.90 (9)	C15—C14—H14A	109.9
C8—C4—C5	107.90 (13)	C15—C14—H14B	109.9
C8—C4—C13	126.06 (14)	C16—C15—C14	121.01 (15)
C13—C4—Mo1	122.25 (10)	C16—C15—C20	118.16 (15)
C4—C5—Mo1	71.79 (8)	C20—C15—C14	120.50 (15)
C4—C5—C9	126.16 (14)	C15—C16—H16	119.4
C6—C5—Mo1	74.82 (8)	C15—C16—C17	121.15 (16)
C6—C5—C4	108.20 (13)	C17—C16—H16	119.4
C6—C5—C9	125.19 (14)	C16—C17—H17	120.1
C9—C5—Mo1	125.37 (11)	C18—C17—C16	119.82 (17)
C5—C6—Mo1	69.76 (8)	C18—C17—H17	120.1
C5—C6—C7	107.84 (13)	C17—C18—H18	120.1
C5—C6—C10	125.02 (14)	C17—C18—C19	119.80 (16)
C7—C6—Mo1	74.13 (8)	C19—C18—H18	120.1
C7—C6—C10	126.43 (14)	C18—C19—H19	119.9
C10—C6—Mo1	129.10 (11)	C20—C19—C18	120.13 (16)
C6—C7—Mo1	71.08 (8)	C20—C19—H19	119.9
C6—C7—C8	108.39 (13)	C15—C20—H20	119.5
C6—C7—C11	125.66 (14)	C19—C20—C15	120.92 (16)
C8—C7—Mo1	71.18 (8)	C19—C20—H20	119.5
C8—C7—C11	125.60 (14)		

Symmetry code: (i) −*x*+2, −*y*+1, −*z*+1.

### Hydrogen-bond geometry (Å, º)

**Table:**

*D*—H···*A*	*D*—H	H···*A*	*D*···*A*	*D*—H···*A*
C10—H10*A*···O2^i^	0.98	2.54	3.277 (2)	132
C12—H12*B*···O1^ii^	0.98	2.62	3.432 (2)	141
C12—H12*C*···O3^i^	0.98	2.47	3.127 (2)	124
C20—H20···O3^iii^	0.95	2.65	3.579 (2)	167

Symmetry codes: (i) −*x*+2, −*y*+1, −*z*+1; (ii) *x*, *y*−1, *z*; (iii) *x*−1, *y*−1, *z*.

## References

[bb1] Brown, L. C., Ressegue, E. & Merola, J. S. (2016). *Organometallics*, **35**, 4014–4022.

[bb2] Bruno, I. J., Cole, J. C., Edgington, P. R., Kessler, M., Macrae, C. F., McCabe, P., Pearson, J. & Taylor, R. (2002). *Acta Cryst.* B**58**, 389–397. 10.1107/s0108768102003324 12037360

[bb3] Clark, R. C. & Reid, J. S. (1995). *Acta Cryst.* A**51**, 887–897.

[bb4] Clegg, W., Compton, N. A., Errington, R. J. & Norman, N. C. (1988). *Acta Cryst.* C**44**, 568–570.

[bb5] Desiraju, G. R. (2002). *Acc. Chem. Res.* **35**, 565–573. 10.1021/ar010054t 12118996

[bb6] Dolomanov, O. V., Bourhis, L. J., Gildea, R. J., Howard, J. A. K. & Puschmann, H. (2009). *J. Appl. Cryst.* **42**, 339–341.

[bb7] Dong, F., Zhi-Hong, M., Su-Zhen, L., Zhan-Gang, H., Zheng, X.-Z. & Lin, J. (2015). *Chin. J. Inorg. Chem.* **31**, 198–204.

[bb8] DuChane, C. M., Brown, L. C., Dozier, V. S. & Merola, J. S. (2018). *Organometallics*, **37**, 530–538.

[bb9] Gould, R. O., Barker, J. & Kilner, M. (1988). *Acta Cryst.* C**44**, 461–463.

[bb10] Groom, C. R., Bruno, I. J., Lightfoot, M. P. & Ward, S. C. (2016). *Acta Cryst.* B**72**, 171–179. 10.1107/S2052520616003954 PMC4822653 27048719

[bb11] Ma, Z., Wang, N., Guo, K., Zheng, X. & Lin, J. (2013). *Inorg. Chim. Acta*, **399**, 126–130.

[bb12] Ma, Z.-H., Wang, H., Han, Z.-G., Zheng, X.-Z. & Lin, J. (2015). *Chin. J. Struct. Chem.* **34**, 931–937.

[bb13] Ma, Z.-H., Zhao, M.-X., Li, F., Liu, X.-H. & Lin, J. (2009). *Chin. J. Inorg. Chem.* **25**, 1699–1702.

[bb14] Ma, Z.-H., Zhao, M.-X., Lin, L.-Z., Han, Z.-H. & Lin, J. (2010). *Chin. J. Inorg. Chem.* **26**, 1908–1911.

[bb15] Macrae, C. F., Bruno, I. J., Chisholm, J. A., Edgington, P. R., McCabe, P., Pidcock, E., Rodriguez-Monge, L., Taylor, R., van de Streek, J. & Wood, P. A. (2008). *J. Appl. Cryst.* **41**, 466–470.

[bb16] Piou, T., Romanov-Michailidis, F., Romanova-Michaelides, M., Jackson, K. E., Semakul, N., Taggart, T. D., Newell, B. S., Rithner, C. D., Paton, R. S. & Rovis, T. (2017). *J. Am. Chem. Soc.* **139**, 1296–1310. 10.1021/jacs.6b11670 PMC5545783 28060499

[bb17] Rigaku OD (2018). *CrysAlis PRO*. Rigaku Oxford Diffraction, Yarnton, UK.

[bb18] Sheldrick, G. M. (2015*a*). *Acta Cryst.* A**71**, 3–8.

[bb19] Sheldrick, G. M. (2015*b*). *Acta Cryst.* C**71**, 3–8.

[bb20] Steiner, T. & Desiraju, G. R. (1998). *Chem. Commun.* pp. 891–892.

[bb21] Taylor, R. (2016). *Cryst. Growth Des.* **16**, 4165–4168.

[bb22] Werner, H. (2012). *Angew. Chem. Int. Ed.* **51**, 6052–6058.

